# Primary pulmonary meningioma mimicking lung metastatic tumor: a case report

**DOI:** 10.1186/s13019-018-0787-5

**Published:** 2018-10-01

**Authors:** Ji-Zhuang Luo, Cheng Zhan, Xiang Ni, Yu Shi, Qun Wang

**Affiliations:** 1 Department of Thoracic Surgery, Zhongshan Hospital, Fudan University, Shanghai, 200032 People’s Republic of China; 2 Department of Pathology, Zhongshan Hospital, Fudan University, Shanghai, 200032 People’s Republic of China

**Keywords:** Primary pulmonary meningioma, PPM, Progesterone receptor

## Abstract

**Background:**

Primary pulmonary meningioma (PPM) is an extremely rare benign tumor. Previous reports indicated that CT features of PPM are single, solid, well-demarcated, homogeneous mass. In this study, we report a case of PPM with atypical CT features.

**Case presentation:**

A 65-year-old female presents to clinic with 1-week acute upper respiratory tract infection. Her chest CT scan revealed a 25–29 mm, round-like, heterogeneous lobulated solitary pulmonary nodule in the right lower lobe. Based on the microscopic features and a wide range of immunohistochemical examinations including vimentine, progesterone receptor (PR), CD34 and S100, the mass was diagnosed as PPM after surgery.

**Conclusion:**

PPM is a rare disease, CT features of PPM could be heterogeneous and lobulated. Expression of vimentine, PR, CD34 and S100 helps to diagnosis of PPM.

## Background

Primary pulmonary meningioma is an extremely rare tumor with uncertain origin. Most PPMs are asymptomatic, grow slowly, and present a benign biological behavior [1]. Histologically, the tumor cells usually show whorls of spindle cells interspersed with abundant psammoma bodies, and positive for vimentin, and EMA (epithelial membrane antigen) [2]. The typical CT features of PPM are single, solid, well-demarcated, homogeneous mass, which was difficult to distinguish with several benign and malignant lung tumors. We report here a 65-year-old female PPM patient with relatively atypical CT findings. We also performed a wide range of IHC examinations for differential diagnosis.

## Case presentation

**Fig. 1:**
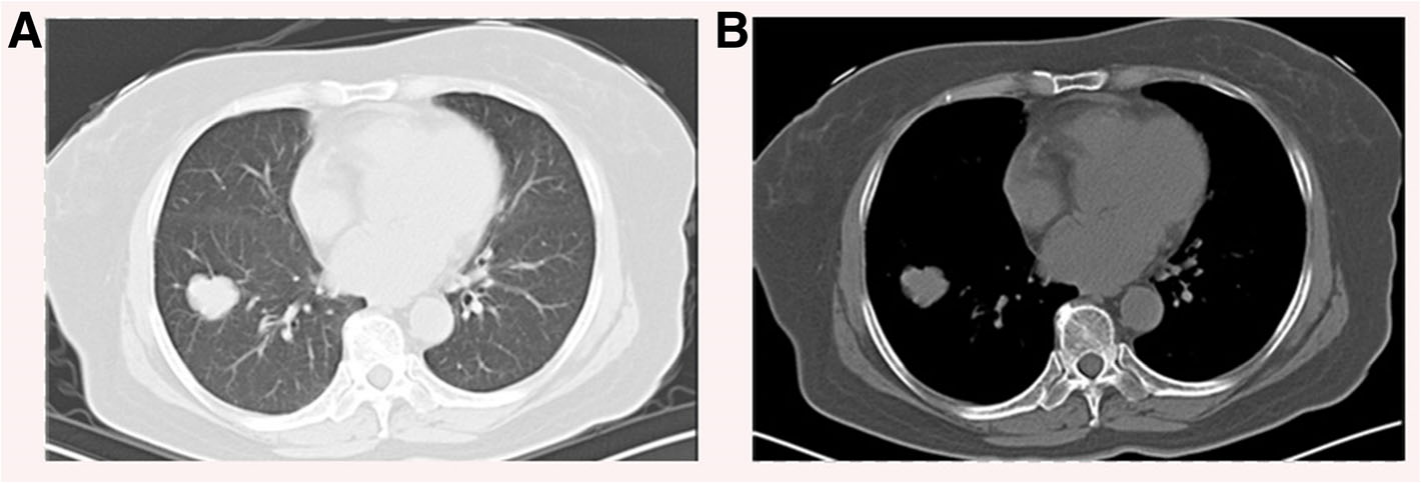
Chest CT scan showing a nodule in the right lower lung field. **a** Lung window. **b** Mediastinal window

A 65-year-old female presents to clinic with 1 week cough. She denies fever, dyspnea, chest pain or constitutional symptoms. She had a history of type II diabetes but with an inadequate controlled serum glucose level for 10 years. Her neutrophils, C-reaction protein (CRP), procalcitonin and tumor markers such as carcinoembryonic antigen (CEA), neuron-specific enolase (NSE), squamous cell carcinoma antigen (SCC) were all in normal range. Her T-SPOT.TB assay examination were negative. A CT scan of her chest reveals a 25–29 mm, round-like, heterogeneous lobulated solitary pulmonary nodule without showing any cavity lesions or calcification, in the peripheral right lower lobe. No obvious enlarged hilar or mediastinal lymph nodes were observed (Fig. 1). Preoperative brain magnetic resonance imaging (MRI) scans showed negative findings. The patient hadn’t received PET/CT scan for economic reasons.

**Fig. 2:**
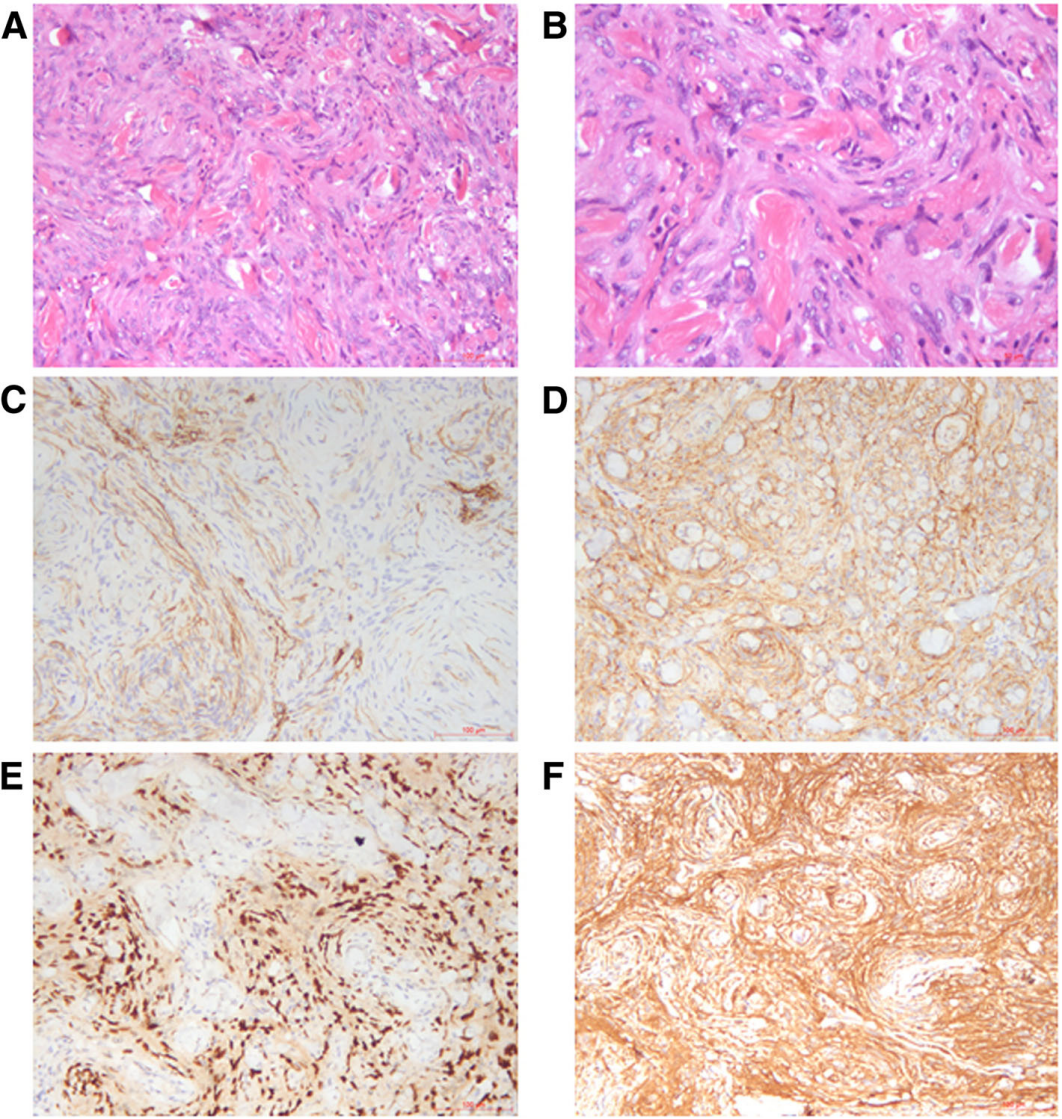
Histological section of the tumor demonstrating spindle cells with a whorl pattern (**a** [20×] and **b** [40×]). On immunohistochemistry the tumor cells stained positive with VIM (**c** [20×]), EMA (**d** [20×]), PR (**e** [20×]) and CD 34 (**f** [20×])

The patient received lobectomy of right lower lobe and systemic mediastinal lymph nodes dissection. A gross examination revealed a 3.5 × 2 × 1.5 cm, well-demarcated, white-pan solid mass. Microscopically, the tumor was composed of spindle cells with a whorl pattern. No atypical cells and mitotic figures were observed (Fig. 2a and b). Immunohistochemically, the tumor cells were positive for vimentine, EMA, PR (progesterone recepters), CD34, D2–40, S100 (focal) and 2% tumor cells positive for Ki-67 (Fig. 2c–f). The tumor cells were negative for synaptophysin, SMA (smooth muscle actin), TTF-1 (thyroid transcription factor-1), Chromogranin A, desmin, GFAP, WT-1 (Wilm’s tumor gene-1), HBME-1 (Hector Battifora mesothelial epitope-1). Based on the above findings, the tumor was diagnosed as primary pulmonary meningioma (PPM).

## Discussion

Primary pulmonary meningioma is an extremely uncommon lung neoplasm which was first described in 1981 by Erlandson. To date, no more than 50 cases have been reported in the English literature. The origin of PPMs remains uncertain, intrathoracic differentiation of meningocytes, ectopic embryonic remnants, minute pulmonary meningothelial-like node may account for the pathogenesis of PPM. Macroscopically, the tumors are solitary, well circumscribed with a yellow-tan to grey cut surface. Histologically, the classical features of PPM showed whorls of spindle cells interspersed with abundant psammoma bodies. Tumor cells were positive for vimentine and EMA, and were negative for SMA, TTF-1, Chromogranin A, synaptophysin. Additionally, we found that PPM cells also expressed PR, CD34 and focally for S100 [3].

The incidence of the disease is slightly higher in women than in men, and the median age is 57 years, with a range of 18–108 years. We report here a 65 year old female with no specific symptoms, no evidence of infection, tuberculosis, no enlargement of hilar and mediastinal lymph nodes, no lesion of extrathoracic organs. However, CT scans of our patient revealed a round-like, heterogeneous lobulated solitary pulmonary mass, possible diagnoses were tuberculoma, metastatic tumor, and benign pulmonary tumors. Previous studies indicated that most PPMs were peripheral, round-shape, well circumscribed and a homogeneous pattern on CT scans. Typical CT features of pulmonary metastases tumors were single or multiple, round, well circumscribed with smooth margin. A halo of ground-glass opacity surrounded the tumor may cause haemorrhage. Others preoperative differential diagnoses including sclerosing hemangioma, hyalinizing granuloma, intrapulmonary fibrous tumor and inflammatory pseudotumor, but the final diagnosis relies on pathological examination. Surgical resection was the curable treatment method, and the prognosis of PPM patients were excellent. Matteo and colleagues [4] summarized the surgical procedures of all 22 PPM patients with a definitive evidence of central nervous system negative for meningioma. There were 12 patients received lobectomy, nine received wedge resection and one receive pneumonectomy. Twenty patients were alive and disease free after a median follow-up time of 30 (5–96) months, which demonstrated that PPM exhibit a benign behavior. No patients had received postsurgical adjuvant therapy. It remains to be observe whether there will be recurrence after surgery.

## Conclusions

PPM is an extremely rare benign tumor. The CT features of PPM can be presented as heterogeneous, lobulated mass. Expression of vimentine, EMA, PR, CD34 and S100 helps to diagnosis of PPM.

## Abbreviations

CEA: Carcinoembryonic antigen
CRP: C-reaction protein
EMA: Epithelial membrane antigen
IHC: Immunohistochemical
MRI: Magnetic resonance imaging
NSE: Neuron-specific enolase
PPM: Primary pulmonary meningioma
PR: Progesterone receptor
SCC: Squamous cell carcinoma antigen
